# Clinical Features and Outcomes in Adult Patients with Autoimmune Encephalitis Requiring Intensive Care: A Retrospective Cohort Study

**DOI:** 10.1007/s12028-025-02374-2

**Published:** 2025-09-16

**Authors:** Lixia Qin, Kexin Chen, Yiwen Zhou, Wei Wang, Wei Lu, Hainan Zhang

**Affiliations:** 1 Department of Neurology, The Second Xiangya Hospital, Central South University, Changsha, Hunan China; 2 Clinical Medical Research Center for Stroke Prevention and Treatment of Hunan Province, Department of Neurology, The Second Xiangya Hospital, Central South University, Changsha, Hunan China

**Keywords:** Autoimmune encephalitis, Intensive care unit, Clinical characteristics, Antibodies, Prognostic prediction model

## Abstract

**Background:**

This study aims to explore the predictors of poor outcomes by analyzing the clinical characteristics and prognosis of adult patients with severe forms of autoimmune encephalitis (AE) requiring intensive care unit (ICU) admission.

**Methods:**

A retrospective analysis was conducted on 134 adult patients diagnosed with definite or possible AE and admitted to the neurology ICU between January 2015 and December 2023. Neurological outcomes at 6 and 12 months were assessed using the modified Rankin scale (mRS). The study further analyzed the relationship between their clinical characteristics, auxiliary examinations, and prognosis.

**Results:**

A total of 134 adult patients with AE requiring ICU admission were included. The 6- and 12-month survival rates were 91.8% and 91.5%, respectively. At 6 months, 72.4% (97 of 134) of patients had favorable outcomes (mRS score ≤ 2), whereas 27.6% (37 of 134) had poor outcomes (mRS score ≥ 3). Compared with the favorable group, patients in the poor outcome group were older (42.92 vs. 30.71 years, *p* = 0.002), had a higher incidence of tumors (24.3% vs. 4.1%, *p* < 0.001), and were more likely to require mechanical ventilation (67.6% vs. 26.8%, *p* < 0.001). They also had lower Glasgow Coma Scale scores on ICU admission (*p* = 0.006), higher Acute Physiology and Chronic Health Evaluation II scores (*p* = 0.006), elevated cerebrospinal fluid glucose (*p* = 0.004) and protein levels (*p* = 0.029), higher autoantibody seronegativity (32.4% vs. 13.4%, *p* = 0.011), lower glucocorticoid use (*p* = 0.038), and longer ICU stays (*p* = 0.031). Multivariate logistic regression identified age (*p* = 0.001), presence of tumor (*p* = 0.03), mechanical ventilation (*p* = 0.025), antibody negativity (*p* = 0.042), and ICU length of stay (*p* = 0.000) as independent predictors of poor prognosis.

**Conclusions:**

These factors may help identify high-risk patients with AE early, enabling timely and targeted interventions to improve outcomes.

## Introduction

Autoimmune encephalitis (AE) is a group of noninfectious, immune-mediated inflammatory disorders of the brain parenchyma, commonly involving the cortex and deep gray matter. It was initially identified in patients with tumor associated with intracellular paraneoplastic antigens (e.g., Hu) [1]. Since the discovery of anti-N-methyl-D-aspartate receptor (NMDA-R) antibody in 2007, a series of autoantibodies targeting neuronal surface or synaptic proteins have been identified, including anti-γ‐amino butyric acid type B receptor (GABA-B-R) antibodies, anti-glutamic acid decarboxylase (GAD) 65 antibodies, anti-metabotropic glutamate receptor (mGluR) antibodies, and anti-α‐amino‐3‐hydroxy‐5‐methyl‐4‐isoxazolepropionic acid receptor (AMPAR) antibodies. Anti-NMDA-R encephalitis remains the most common form of AE; however, a subset of patients lack identifiable antibodies.

The clinical manifestations of AE are diverse, encompassing psychiatric and behavioral abnormalities, seizures, movement disorders, and cognitive decline [2]. Additional symptoms include sleep disturbances, autonomic dysfunction, and peripheral neuropathy. Severe cases require intensive care unit (ICU) management due to uncontrollable psychiatric symptoms, status epilepticus, central hypoventilation, or severe involuntary movements. Studies have shown that 75% of anti-NMDA-R encephalitis cases require ICU care [3]. AE is a treatable yet potentially disabling and life-threatening central nervous system (CNS) inflammatory disorder, particularly in critical ICU patients. ICU admission has been identified as a significant predictor of poor outcomes [4]. However, the clinical features and prognostic factors for critical patients with AE in the ICU remain inconclusive.

This study retrospectively reviewed critical patients with AE admitted to our center over the past decade, describing the clinical characteristics associated with varying outcomes in the ICU. It analyzed prognostic factors linked to different clinical outcomes and established a prognostic prediction model based on clinical characteristics. This model facilitates early identification of critical patients with AE with poor prognoses, enabling earlier comprehensive management and therapeutic interventions to improve patient outcomes.

## Materials and Methods

### Study Design

We conducted a retrospective study of patients diagnosed with AE who were admitted to the ICU of the Neurology Department at the Second Xiangya Hospital of Central South University from January 2015 to June 2024. Follow-up evaluations were conducted at 3, 6, and 12 months after disease onset. The diagnoses of all patients were reevaluated. Patients were included if they fulfilled the following criteria: (1) met the clinical diagnostic criteria for “definite” or “probable” AE proposed by Graus et al. in 2016 [5] or the Chinese Society of Neurology in 2022 [6]; (2) aged ≥ 14 years; and (3) admitted to an adult ICU during the course of the disease. The exclusion criteria were deemed as (1) other potential causes of symptoms, including CNS infections, septic encephalopathy, metabolic encephalopathy, and mitochondrial encephalopathy; (2) a history of severe cardiovascular, hematological, or urinary system diseases; (3) previous or current major systemic or neurological diseases or brain injuries affecting brain structure or function; (4) inability to complete the 6-month follow-up; and (5) an ICU length of stay of 24 h or less. The study was approved by the Ethics Committee of the Second Xiangya Hospital of Central South University (No. LYF20240199).

### Antibodies Detection

Antibodies against NMDA-R, leucine-rich glioma-inactivated protein 1 (LGI1), contactin associated protein 2 (CASPR2), GABA-B-R, AMPAR1, AMPAR2, dipeptidyl-peptidase-like protein (DPPX), IgLON5, GAD65, glycine receptor 1 (GlyR1), mGluR, D2 dopamine receptor (D2R), GABA-A-R, amphiphysin, neurexin-3a, adenylate kinase 5 (AK5), Kelch-like protein-11 (KLHL11), myelin oligodendrocyte glycoprotein (MOG), glial fibrillar acidic protein (GFAP), Hu, Yo, Ri, CV2, and Ma2 were tested in both serum and cerebrospinal fluid (CSF). The primary testing method was the cell-based assay (CBA), including both fixed and live CBAs. Some samples were additionally evaluated using the tissue-based assay, and paraneoplastic antibodies were assessed by immunoblotting.

### Data Collection and Outcomes

We collected demographic data, primary symptoms, CSF analysis results, imaging findings, electroencephalogram (EEG) results, treatment protocols, and clinical outcomes at 3, 6, and 12 months following disease onset. Abnormal brain magnetic resonance imaging (MRI) patterns were mainly defined as limbic, cortical/subcortical, striatal, diencephalic, brainstem, encephalomyelitis, and meningoencephalitis according to the 2016 AE clinical criteria by Graus et al. [5]. Clinical outcomes were assessed using the modified Rankin scale (mRS), where an mRS score ≤ 2 indicated a favorable outcome, and an mRS score ≥ 3 indicated an unfavorable outcome. The evaluations were conducted by an experienced operator (L.Q.) using a structured interview format to ensure consistency and homogeneity in the assessments. Whenever feasible, follow-ups were performed at 3, 6, and 12 months after disease onset, with a minimum follow-up duration of 6 months.

### Statistical Analysis

Parametric variables were expressed as mean ± standard deviation, whereas nonparametric variables were expressed as median (interquartile range [IQR]). Two-group comparisons of normally distributed continuous variables were performed using the *t*-test, whereas the Mann–Whitney *U*-test was used for nonnormally distributed continuous variables. Categorical variables were compared using the χ^2^ test. Variables with a *p* value < 0.1 in univariate logistic regression were included in multivariate logistic regression, with variable selection performed using the backward likelihood ratio method. Statistical significance was set at *p* < 0.05. All statistical analyses were conducted using IBM SPSS (v26, Chicago, IL) or R software (v4.2.2).

## Results

### Patient Characteristics

A total of 134 patients with AE (definite AE, 109; probable AE, 25) who were admitted to the ICU and completed the 6-month follow-up evaluation were included in this study, among whom 129 patients completed the 12-month follow-up. Male patients accounted for 51.5% (69 of 134), and the median age at onset was 34.2 years (IQR 21–44.75).

Among all patients, 51.5% (69 of 134) had prodromal infections or psychological stressors, 9.7% had concomitant tumors, and 38.1% required ventilation. Regarding clinical manifestations, the most common symptoms were psychiatric and behavioral abnormalities (68.7%) and seizures/status epilepticus (64.2%), whereas 13.4% of patients presented with movement disorders. MRI was performed in 96.3% of patients, and a pathologic result was obtained in 31%. EEG was performed in 62.7% of patients and showed epileptic discharges in 56.9% of patients. CSF was analyzed in 97% (130 of 134) of the patients; 51.5% (67 of 130) had pleocytosis, and 23.1% (30 of 130) showed an increased total protein content (Table 1).

**Table 1:** Demographic and clinical profile of patients with critical patients with AE in the ICU

Variable	All (*N* = 134)	Good prognosis (*n* = 97)	Poor prognosis (*n* = 37)	*p* value
Age at onset, median (IQR), year	34.20 (21.00–44.75)	30.71 (20.00–38.00)	42.92 (23.00–59.50)	**0.002**
*Sex, n (%)*				
Male	69 (51.5)	49 (50.5)	20 (54.1)	0.71
Female	65 (48.5)	43 (50.0)	22 (45.8)	
Prodromal infection, %	69 (51.5)	52 (53.6)	17 (45.9)	0.43
Tumor, %	13 (9.7)	4 (4.1)	9 (24.3)	**< 0.001**
Ventilation, %	51 (38.1)	26 (26.8)	25 (67.6)	**< 0.001**
*Symptoms, n (%)*				
Psychiatric symptoms	92 (68.7)	69 (71.1)	23 (62.2)	0.32
Seizures/status epilepticus	86 (64.2)	53 (64.9)	23 (62.2)	0.76
Movement disorder	18 (13.4)	11 (11.3)	7 (18.9)	0.25
Others	97 (72.4)	71 (73.2)	26 (70.3)	0.74
*Patients’ characteristics on ICU admission*				
GCS < 8, *n* (%)	21 (15.7)	10 (10.3)	11 (29.7)	**0.006**
APACHE-II score, median (IQR)	8.19 (4.00–12.25)	7.42 (3.50–10.00)	10.19 (6.00–15.00)	**0.006**
*Blood examination, median (IQR)*				
SIRI (PLT*NLR)	2,046.36 (480.44–2,014.61)	1,845.57 (672.58–1,918.54)	2,572.76 (698.11–2,294.47)	0.341
NLR	8.79 (3.33–8.7)	8.03 (3.30–7.90)	10.78 (3.76–11.45)	0.154
PLR	216.19 (126.32–254.82)	209.54 (126.10–247.79)	233.62 (127.86–267.77)	0.491
*CSF analysis, median (IQR)*				
Leukocyte, 10^6^/L	35.46 (2.00–28.50)	36.55 (2.00–30.00)	32.73 (2.00–29.00)	0.53
Lymphocyte, 10^6^/L	26.42 (0.00–25.00)	26.21 (0.00–22.50)	26.92 (1.00–25.50)	0.24
Protein, mg/L	348.07 (137.97–440.38)	308.20 (130.74–375.54)	448.28 (152.00–513.00)	**0.029**
Glucose, mmol/L	3.88 (3.21–4.45)	3.73 (3.20–4.16)	4.25 (3.67–4.68)	**0.004**
*Brain MRI, n (%)*				
Evaluated	129 (96.3)	93 (95.9)	36 (97.3)	0.70
Abnormal MRI	40 (31.0)	29 (31.2)	11 (30.6)	0.95
*EEG, n (%)*				
Evaluated	84 (62.7)	63 (64.9)	21 (56.8)	0.38
Epileptic waves	56 (65.9)	39 (60.9)	17 (81.0)	0.09
*Autoantibodies, n (%)*				
Positive	109 (81.3)	84 (86.6)	25 (67.6)	**0.011**
Negative	25 (18.7)	13 (13.4)	12 (32.4)	
*Treatment*				
Glucocorticoid, *n* (%)	115 (85.8)	87 (89.7)	28 (75.7)	**0.038**
Interval from onset to glucocorticoid treatment, median (IQR), day	20.1 (10.00–25.00)	21.21 (10.00–26.00)	16.64 (10.25–19.00)	0.696
IVIG, *n* (%)	102 (76.1)	76 (78.4)	26 (70.3)	0.33
Interval from onset to IVIG treatment, median (IQR), day	18.66 (8.75–23.25)	20.42 (8.25–26.00)	13.50 (8.50–20.00)	0.156
PE/immunoadsorption, *n* (%)	12 (9.0)	6 (6.2)	6 (16.2)	0.069
Interval from onset to PE or immunoadsorption treatment, median (IQR), day	22.08 (11.50–26.50)	23.5 (9.75–32.75)	20.86 (18.00–27.00)	0.534
Duration ICU stay, median (IQR) day	20.51 (5.00–27.00)	16.06 (4.00–22.50)	32.19 (6.50–41.00)	**0.031**

Statistically significant values (*p* < 0.05) are shown in bold.

Good prognosis: mRS score ≥ 3 at 6 months; poor prognosis: mRS score ≤ 2 at 6 months

*AE* Autoimmune encephalitis, *APACHE-II* Acute Physiology and Chronic Health Evaluation II, *CSF* Cerebrospinal fluid, *EEG* Electroencephalogram, *GCS* Glasgow coma scale, *ICU* Intensive care unit, *IQR* Interquartile range, *IVIG* Intravenous immunoglobulin, *MRI* Magnetic resonance imaging, *mRS* Modified Rankin scale, *NLR* Neutrophil-to-lymphocyte ratio, *PE* Plasma exchange, *PLR* Platelet-to-lymphocyte ratio, *SIRI* Systemic inflammation response index.

**Fig. 1:**
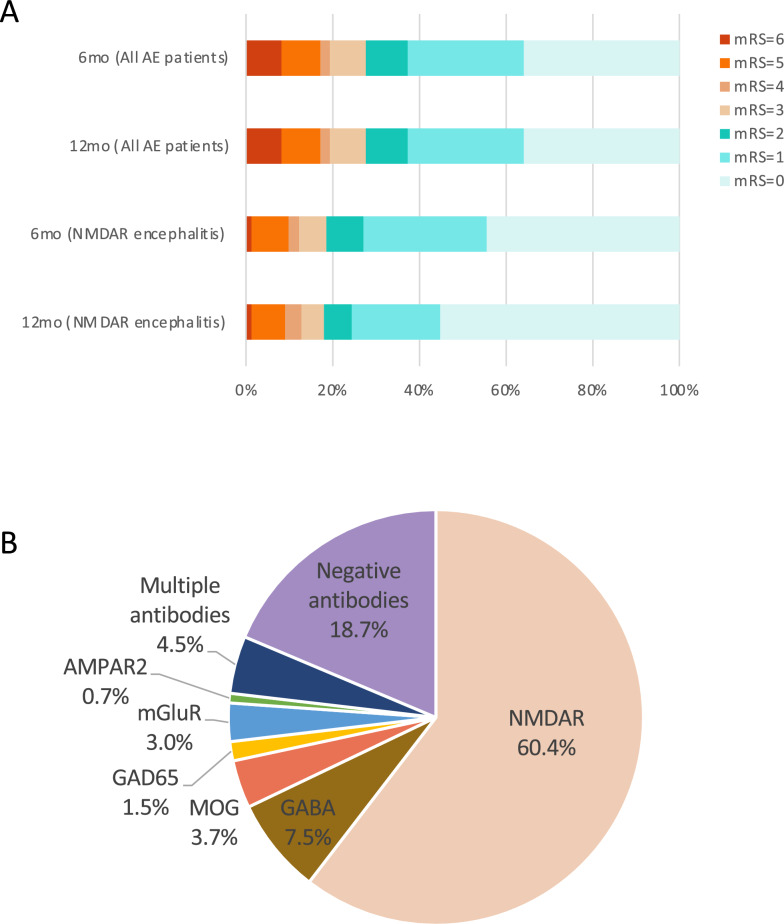
**a** Distribution of modified Rankin scale (mRS) scores at different time points. Distribution of mRS scores at 6 and 12 months after onset for all patients and patients with anti-NMDA-R encephalitis, respectively. **b** Antibody subtypes in our cohort. A total of 81 cases (59.1%) were positive for anti-NMDA-R antibodies, 10 cases (7.5%) were positive for anti-GABA-B-R antibodies, 5 cases (3.7%) were positive for anti-MOG antibodies, 2 cases (1.5%) were positive for anti-GAD65 antibodies, 4 cases (3.0%) were positive for anti-mGluR antibodies, 1 case (0.7%) was positive for anti-AMPAR antibodies, 6 cases (4.5%) were positive for multiple antibodies, and 25 cases (18.7%) were antibody negative. AE autoimmune encephalitis

Neuronal autoantibodies were examined in all of the patients in both serum and CSF. Positive antibodies were detected in 109 of 134 patients. Anti-NMDA-R antibodies were the most commonly identified, detected in 81 patients (60.4%). At 6 months after onset, 15 patients with anti-NMDA-R encephalitis had poor outcomes (20.0%), including 5 with an mRS score of 3, 2 with an mRS score of 4, 7 with an mRS score of 5, and 1 death (Fig. 1A). Psychiatric and behavioral abnormalities were the most prevalent clinical manifestation among patients with anti-NMDA-R encephalitis, occurring in 80.2% of cases, with a relatively low rate of poor prognosis (18.5%). Other antibody types included anti-GABA-B-R (10 cases), anti-MOG (5 cases), anti-GAD65 (2 cases), anti-mGluR (4 cases), and anti-AMPAR (1 case). Additionally, 6 patients (3.9%) had multiple autoantibodies: 1 patient coexpressed anti-NMDA-R and anti-GABA-B-R antibodies, 1 patient coexpressed anti-NMDA-R and anti-GABA-A-R antibodies, three had anti-NMDA-R and anti-MOG antibodies, and one patient was positive for both anti-MOG and anti-GlyR antibodies (Fig. 1B and Table 2).

**Table 2:** Clinical characteristics of ICU critically ill patients with AE of different antibody types

Autoantibody subtype	*n*	Prodromal infection, *n* (%)	Symptoms, *n* (%)				Tumor, *n* (%)	Poor prognosis, *n* (%)
			Psychiatric symptoms	Seizures	Movement disorder	Others		
NMDA-R	81	38 (46.9)	65 (80.2)	49 (60.5)	15 (18.5)	63 (77.8)	6 (7.4)	15 (18.5)
GABA-B-R	10	5 (50)	6 (60)	8 (80)	0 (0)	5 (50)	3 (30)	6 (60)
MOG	5	3 (60)	0 (0)	3 (60)	0 (0)	4 (80)	0 (0)	2 (40)
GAD65	2	1 (50)	0 (0)	2 (100)	0 (0)	2 (100)	0 (0)	1 (50)
mGluR	4	1 (25)	3 (75)	3 (75)	0 (0)	1 (25)	0 (0)	0 (0)
AMPAR2	1	0 (0)	1 (100)	0 (0)	0 (0)	1 (100)	1 (100)	0 (0)
Multiple antibodies	6	3 (50)	4 (66.7)	3 (50)	0 (0)	4 (66.7)	0 (0)	1 (16.7)
Negative antibodies	25	18 (72)	13 (52)	18 (72)	3 (12)	17 (68)	3 (12)	12 (48)

Statistically significant values (*p* < 0.05) are shown in bold.

*AE* Autoimmune encephalitis, *ICU* Intensive care unit

### Potential Prognostic Factors and Prognosis Prediction Model

The 6-month survival rate was 91.8% (123 of 134), and the 12-month survival rate was 91.5% (118 of 129). The distribution of mRS scores at each time point is shown in Fig. 1A. All patients were categorized into two groups based on their mRS scores at 6 months after disease onset: the favorable outcome group (mRS score ≤ 2, n = 97, 72.4%) and the poor outcome group (mRS score ≥ 3, n = 37, 27.6%). Compared to the favorable outcome group, patients in the poor outcome group were older at disease onset (42.92 vs. 30.71 years, *p* = 0.002), had a higher incidence of associated tumors (24.3% vs. 4.1%, *p* < 0.001), and were more likely to require mechanical ventilation (67.6% vs. 26.8%, *p* < 0.001) (Table 1). In addition, a greater proportion of patients in the poor outcome group had low Glasgow Coma Scale (GCS) scores upon ICU admission (29.7% vs. 10.3%, *p* = 0.006), higher Acute Physiology and Chronic Health Evaluation II (APACHE-II) scores (10.19 vs. 7.42, *p* = 0.006), elevated CSF glucose levels (4.25 mmol/L vs. 3.73 mmol/L, *p* = 0.004), and increased CSF protein concentrations (448.28 mg/L vs. 308.20 mg/L, *p* = 0.029). Moreover, the seronegativity rate for autoantibodies was higher in the poor outcome group (32.4% vs. 13.4%, *p* = 0.011), glucocorticoid use was less frequent (75.7% vs. 89.7%, *p* = 0.038), and ICU length of stay was significantly longer (32.19 days vs. 16.06 days, *p* = 0.031).

Univariate logistic regression analysis revealed that age (*p* < 0.001), presence of tumors (*p* = 0.002), ventilation (*p* < 0.001), GCS score at ICU admission (*p* = 0.008), APACHE-II score (*p* = 0.013), CSF glucose level (*p* = 0.005), antibody positivity (*p* = 0.014), use of glucocorticoids (*p* = 0.043), and ICU length of stay (*p* = 0.005) were significantly associated with prognosis (Table 3). Variables with a *p* value < 0.1 in the univariate analysis were included in the multivariate logistic regression model to assess their predictive value using receiver operating characteristic curve analysis. The results indicated that age (*p* = 0.001), presence of tumors (*p* = 0.03), mechanical ventilation (*p* = 0.025), antibody negativity (*p* = 0.042), and ICU length of stay (*p* = 0.00) were independent prognostic factors (Table 4). A prognostic prediction model for adult patients with severe AE was established by combining these four indicators, yielding the highest area under the curve.

**Table 3:** Univariate analysis to identify indicators with significant differences between good and poor prognosis groups

Variable	B	OR (95% CI)	*p* value
Sex	− 0.142	0.87 (0.41–1.85)	0.71
Age at onset, year	0.042	1.04 (1.02–1.07)	< 0.001^**^
Prodromal infection	− 0.31	0.74 (0.34–1.57)	0.43
Psychiatric symptoms	− 0.41	0.67 (0.30–1.48)	0.32
Seizures/status epilepticus	− 0.12	0.89 (0.41–1.94)	0.76
Movement disorder	0.60	1.82 (0.65–5.13)	0.26
Other symptoms	− 0.14	0.87 (0.38–2.00)	0.74
Tumor	2.01	7.47 (2.14–26.12)	0.002^**^
Ventilation	1.74	5.69 (2.50–12.94)	< 0.001^**^
GCS < 8	− 1.30	0.27 (0.10–0.71)	0.008^**^
APACHE-II score	0.09	1.09 (1.02–1.17)	0.013^*^
NLR	0.011	1.01 (0.99–1.04)	0.35
PLR	0.001	1.00 (1.00–1.00)	0.44
SIRI (PLT*NLR)	0.00	1.00 (1.00–1.00)	0.32
Leukocyte count in CSF	− 0.001	1.00 (0.99–1.00)	0.78
Lymphocyte count in CSF	0.00	1.00 (0.99–1.01)	0.94
Glucose level in CSF	0.65	1.92 (1.22–3.02)	0.005^**^
Protein level in CSF	0.001	1.00 (1.00–1.00)	0.07
Epileptic waves	1.002	2.72 (0.82–9.04)	0.101
Positive antibodies	− 1.13	0.32 (1.13–0.80)	0.014^*^
Glucocorticoid	− 1.03	0.36 (0.13–0.97)	0.043^*^
Interval from onset to glucocorticoid treatment	− 0.02	0.98 (0.95–1.01)	0.24
IVIG	− 0.43	0.65 (0.28–1.54)	0.33
Interval from onset to IVIG treatment	− 0.04	0.96 (0.92–1.01)	0.094
PE/immunoadsorption	1.08	2.94 (0.88–9.77)	0.08
Interval from onset to PE or immunoadsorption treatment	− 0.012	0.99 (0.92–1.06)	0.76
Duration ICU stay	0.02	1.02 (1.01–1.04)	0.005^**^

Statistically significant values (*p* < 0.05) are shown in bold.

*APACHE-II* Acute Physiology and Chronic Health Evaluation II, *CI* Confidence interval, *CSF* Cerebrospinal fluid, *GCS* Glasgow Coma Scale, *ICU* Intensive care unit, *IVIG* Intravenous immunoglobulin, *NLR* Neutrophil-to-lymphocyte ratio, *OR* Odds ratio, *PE* Plasma exchange, *PLR* Platelet-to-lymphocyte ratio, *SIRI* Systemic inflammation response index

^*^p ≤ 0.05; **p ≤ 0.01

**Table 4:** Multiple logistic regression analysis for prognostic model

Variable	B	OR (95% CI)	*p* value
Age at onset	0.046	1.047 (1.019–1.077)	0.001
Tumor	1.694	5.443 (1.176–25.199)	0.03
Ventilation	1.108	3.03 (1.06–8.66)	0.025
Positive antibodies	− 1.259	0.284 (0.095–0.851)	0.042
Duration ICU stay	0.02	1.02 (1.001–1.039)	0.00

Statistically significant values (*p* < 0.05) are shown in bold.

*APACHE-II* Acute Physiology and Chronic Health Evaluation II, *CI* Confidence interval, *CSF* Cerebrospinal fluid, *ICU* Intensive care unit, *IVIG* Intravenous immunoglobulin, *OR* Odds ratio

## Discussion

This study describes the clinical characteristics of critically ill patients with AE admitted to our neurologic ICU over the past decade. It analyzes prognostic factors associated with different clinical outcomes, identifying age, presence of tumors, ventilation, GCS score at ICU admission, APACHE-II score, CSF glucose level, antibody positivity, use of glucocorticoids, and ICU length of stay as factors associated with prognosis. Among these, age, ventilation, presence of tumors, antibody positivity, and ICU length of stay were independent predictors of prognosis.

Previous studies have reported a poor prognosis rate of 23.6% for ICU-treated patients with AE [7], which is comparable to the 27.6% observed in our center. Consistent with previous reports, our findings showed that older age was associated with poorer outcomes [7–9]. We speculate that this may be due to delays in diagnosis and treatment, more severe clinical manifestations, and systemic complications [10, 11]. Therefore, in clinical practice, it is important to emphasize early diagnosis and strict monitoring for systemic complication.

A survival analysis conducted in 2017 on ICU-treated patients with AE revealed that the most common reasons for ICU admission were seizures (26%) and delirium (15%), with a 1-year survival rate of 82%. Patients who died were more likely to have concomitant tumors (*p* < 0.041) or require respiratory support (*p* = 0.004) [12]. A 2016 study from the Mayo Clinic included 25 ICU patients with AE [10]. At the final follow-up, the mean mRS score was 3. Ten patients had died, six had mild disability, three had moderate cognitive impairment, and six had dementia. Additionally, other studies have indicated that poor neurological outcomes are associated with mechanical ventilation (odds ratio (OR) 6.28 [95% confidence interval (95% CI) 2.71–15.61]), tracheostomy (OR 6.26 [95% CI 2.68–15.73]), tumors (OR 3.73 [95% CI 1.35–11.57]), sepsis (OR 4.54 [95% CI 1.99–10.43]), or autonomic dysfunction (OR 2.91 [95% CI 1.24–7.3]) [13]. Compared to these studies, the 1-year survival rate of our patients was slightly higher (91.5%). Similarly, previous studies showed that patients with poor prognosis had a higher proportion of tumors (*p* = 0.002) and mechanical ventilation (*p* < 0.01) [12]. Furthermore, our findings revealed that patients with poor prognosis had higher APACHE-II scores. Although APACHE-II is not specific to AE, it reflects the severity of critical illness. In severe AE cases, a high APACHE-II score may lead to the underlying AE diagnosis being overlooked. In such situations, the initial clinical symptoms, brain MRI, EEG, lumbar puncture, and antibody testing are particularly important for accurate diagnosis. Investigating AE only based on the patients’ presentation is more of a challenge when investigations such as brain MRI, EEG, and lumbar puncture are not practical.

Regarding CSF analysis, one study demonstrated that decreased CSF leukocyte count was an independent predictor of good prognosis in ICU patients with anti-NMDA-R encephalitis [4]. Another study on the long-term prognosis of AE revealed CSF pleocytosis was associated with a lower likelihood of good recovery [11]. However, our study did not find an association between CSF leukocyte count and prognosis. Instead, we observed higher levels of CSF glucose and protein in patients with poor prognosis, with CSF protein being an independent prognostic factor. These findings warrant further investigation. The role of status epilepticus as a risk factor for poor prognosis in acute encephalitis remains controversial [8, 14, 15]. A multicenter observational study in 2017 found no association between status epilepticus and prognosis in ICU patients with anti-NMDA-R encephalitis [8]. Similarly, our results do not support seizures or status epilepticus as predictors of poor prognosis in critically ill ICU patients with AE.

Previous studies have shown that common antibody types in ICU patients with AE include NMDA-R, GABA, CASPR2, LGI1, VGKCC, Ma1/Ma2, CV2/CRPM, and AMPAR [7, 10, 12]. A 2025 study on the long-term prognosis of AE reported the most common antibodies in the order of LGI1, GAD65, NMDA-R [11]. In our study, the most frequent antibodies were NMDA-R, GABA-B-R, MOG, and mGluR. This discrepancy may be explained by differences in patient selection: their cohort included all AE cases, whereas ours focused exclusively on severe cases. In addition, our center frequently detected antibodies such as MOG (5 cases), GAD65 (2 cases), and mGluR (4 cases). Neuronal autoantibodies were detected in 109 cases (81.3%), a higher rate than previous studies (85 of 120) [13], likely reflecting advancements in diagnostic techniques. Some studies suggest that antibodies against cell-surface antigens (e.g., NMDA-R, GABA-R, AMPAR, mGluR5, LGI1) are associated with better immunotherapy responses and clinical outcomes compared to antibodies against intracellular antigens (e.g., Hu, CV2, Ma2) [16]. However, other studies have found no association between antibody type and prognosis [13]. Due to the limited sample size of non-NMDA-R antibody cases in our study and the predominance of cell-surface antibodies, we did not analyze prognosis based on antibody classification. A prospective study of probable antibody-negative AE reported favorable outcomes in most patients [17]. Interestingly, in our cohort, seronegative patients with AE had poorer clinical outcomes (48%), likely because our study included only critically ill patients with AE. We speculate that seronegative patients with AE may harbor intracellular antibodies, which are less responsive to immunotherapy. Thus, clinical outcomes may be more closely related to antibody type than to the mere presence of detectable antibodies.

Previous studies found that early immunotherapy is a well-established predictor of good outcomes in anti-NMDA-R encephalitis [3, 8, 18]. This underscores the importance of initiating early immunotherapy in critically ill patients with AE as soon as this diagnosis is suspected and infectious causes are excluded. Our study found that glucocorticoids were used more frequently in the good prognosis group, suggesting that systemic conditions in the poor prognosis group may have affected glucocorticoid administration. Although the poor prognosis group initiated intravenous immunoglobulin and plasma exchange/immunoadsorption earlier, this did not improve outcomes, indicating that other factors may contribute to poor prognosis.

We acknowledge several limitations. First, this was a single-center study with a relatively small sample size. Larger cohorts may yield more accurate results and enable prognostic analysis of different antibody types. Second, this study included only a subset of critical clinical indicators and did not consider second-line immunotherapy or long-term outcomes such as relapse. Third, prognosis was assessed using the mRS alone, which may be subject to subjectivity. However, evaluations were conducted by experienced professionals to minimize bias. Moreover, a variety of residual symptoms (cognitive difficulty, seizures, depression and sleep disorders) may not have been sensitively detected using the mRS. Fourth, in this study, we primarily used the CBA method. Although CBA offers high sensitivity and specificity, fixed CBAs do not routinely provide titers and may yield false negative results. Aside from the Euroimmun CBA, additional testing was limited, which may have constrained the comprehensiveness of antibody detection. Finally, the lack of a validation cohort necessitates further assessment of this model’s effectiveness in independent clinical cohorts.

## Conclusions

In conclusion, this study provides a clinical prognostic risk model for ICU patients with AE. We recommend considering factors such as age, mechanical ventilation, comorbidities, and antibodies in clinical practice to assess prognosis. Early comprehensive management and intervention may improve patient outcomes.
